# Determination of minimum inhibitory and minimum bactericidal concentrations of Brazilian strains of *Leptospira* spp. for streptomycin sulphate

**DOI:** 10.1017/S0950268820002228

**Published:** 2020-09-25

**Authors:** Bruno Ribeiro Rocha, Gabriel Mendes de Souza Martins, Breno Cayeiro Cruz, Walter Lilenbaum

**Affiliations:** 1 Universidade Federal Fluminense, Niterói, RJ, Brazil; 2 Faculdade de Medicina de Petrópolis/Faculdade Arthur Sá Earp Neto, Petrópolis, RJ, Brazil; 3 Ourofino Saúde Animal Ltda, Ribeirão Preto, Brazil

To the Editor,

In 2018, our group published a paper reporting on the reduced antimicrobial susceptibility of *Leptospira* strains of animal origin in this prestigious journal [1]. Meanwhile, new strains were characterised, enlarging the epidemiological profile of leptospirosis in our scenario. We followed the same methodology of the previous paper and tested them against streptomycin sulphate. In this context, the goal of this letter is to enlarge and update the knowledge about the susceptibility of leptospiral strains against streptomycin sulphate.

The current study determined the minimum inhibitory and bactericidal concentrations (MIC and MBC) of streptomycin sulphate, in comparison with six local strains of serogroups Sejroe, Icterohaemorrhagiae, Grippotyphosa and Pomona, belonging to species *Leptospira interrogans*, *L. santarosai* and *L. kirschneri* (Table 1). Strains were maintained in liquid nitrogen, belonging to the Bacteria Collection of Veterinary Interest of the Universidade Federal Fluminense, Rio de Janeiro, Brazil (http://labv.uff.br/ccbvet/#).

**Table 1.** Minimum inhibitory and bactericidal concentrations (MIC and MBC) of streptomycin sulphate in six selected local strains of *Leptospira*

Strain	Species	Serogroup	Clinical sample	MIC (μg/ml)	MBC (μg/ml)
OV5	*L. interrogans*	Sejroe	Vaginal fluid	0.39	1.56
61H	*L. interrogans*	Pomona	Urine	3.13	3.13
3759	*L. kirschneri*	Pomona	Urine	0.78	3.13
U291	*L. santarosai*	Grippotyphosa	Urine	3.13	25
CAP 5940	*L. kirschneri*	Icterohaemorrhagiae	Urine	1.56	1.56
M9/99	*L. interrogans*	Icterohaemorrhagiae	Kidney	3.13	3.13

In this study, MIC values of streptomycin across the different *Leptospira* strains ranged from 0.39 to 3.13 μg/ml, with MBCs ranging from 1.56 to 25 μg/ml (Table 1). These results showed a wide variation in the susceptibility of the strains against streptomycin, not only regarding MIC, but also MBC. The obtained outcomes are consistent with the values observed in other studies such as Correia *et al*. [1] and Liegeon *et al*. [2], and could define all studied strains as sensitive to streptomycin. These findings validate the usage of streptomycin in field conditions, a fact that can be considered positive, since this is the most common antibiotic used for the treatment of leptospirosis in livestock, particularly ruminants.

## Data Availability

Data are available on request from the authors.
